# Acute communication between microglia and other immune cells in the anti‐Aβ antibody injected cortex

**DOI:** 10.1002/alz.090982

**Published:** 2025-01-03

**Authors:** Kate E. Foley, Katelynn E. Krick, Erica M. Weekman, Donna M. Wilcock

**Affiliations:** ^1^ Indiana University School of Medicine/Stark Neurosciences Research Institute, Indianapolis, IN USA; ^2^ Indiana University School of Medicine/ Stark Neurosciences Research Institute, Indianapolis, IN USA; ^3^ Indiana University School of Medicine, Stark Neurosciences Research Institute, Department of Neurology, Indianapolis, IN USA

## Abstract

**Background:**

Anti‐Aβ immunotherapy use to treat Alzheimer’s disease is on the rise. While anti‐Aβ antibodies provide hope in targeting Aβ plaques in the brain, a major side effect of amyloid‐related imaging abnormalities (ARIA) persists with no known cause. As severe ARIA is typically seen within the first few infusions in human clinical trials, we sought to identify acute effects of anti‐Aβ antibody on brain.

**Method:**

To determine cellular changes due to anti‐Aβ antibody exposure, we intracranially injected 14mo APP male and female mice with anti‐Aβ IgG1 (6E10) or control IgG1 into the cortex. After 24hrs or 3days, we harvested the cortex and performed a glial cell enriched preparation for single cell sequencing. Cell types, proportions, and cell‐to‐cell signaling was evaluated between the two injection conditions and two acute timepoints.

**Result:**

We identified 23 unique cell clusters including microglia, astrocytes, endothelial cells, neurons, oligos/OPCs, immune cells, and unknown. The anti‐Aβ antibody injected cortices revealed more ligand‐receptor communications between cell types, as well as stronger communications at 24hrs. At 3days, while there were more L‐R communications for the anti‐Aβ antibody condition, the strength of these connections was stronger in the control IgG condition. Specific pathways such as CD48 and PD‐L1 were enriched in the anti‐Aβ antibody condition, but not the control IgG condition in microglia‐to‐microglia communication. We also found evidence of an initial and strong communication emphasis in microglia‐to‐peripheral immune cells at 24hrs, specifically in the TGFβ signaling pathway. Additionally, we found that peripheral immune cells that were exposed to anti‐Aβ antibody failed to signal via the TNF pathway, which was strongly enriched in the control IgG condition at both timepoints.

**Conclusion:**

We identify several pathways that differ between anti‐Aβ antibody and control IgG injections at acute timepoints. These data lay the groundwork for understanding the brain’s unique response to anti‐Aβ antibody and its predisposition to ARIA.

